# Predicting the risk for colorectal cancer with personal characteristics and fecal immunochemical test

**DOI:** 10.1097/MD.0000000000010529

**Published:** 2018-05-04

**Authors:** Wen Li, Li-Zhong Zhao, Dong-Wang Ma, De-Zheng Wang, Lei Shi, Hong-Lei Wang, Mo Dong, Shu-Yi Zhang, Lei Cao, Wei-Hua Zhang, Xi-Peng Zhang, Qing-Huai Zhang, Lin Yu, Hai Qin, Xi-Mo Wang, Sam Li-Sheng Chen

**Affiliations:** a Department of Epidemiology, Tianjin Colorectal and Anal Disease Research Institute; b Department of Gastroenterology, Tianjin Union Medical Center; c Non-Communicable Disease Control and Prevention, Tianjin Centers for Disease Control and Prevention; d Department of Gastroenterology, Tianjin Nankai Hospital, Tianjin, P.R. China; e School of Oral Hygiene, College of Oral Medicine, Taipei Medical University, Taiwan.

**Keywords:** colorectal cancer, fecal immunochemical test, prediction, screening

## Abstract

We aimed to predict colorectal cancer (CRC) based on the demographic features and clinical correlates of personal symptoms and signs from Tianjin community-based CRC screening data.

A total of 891,199 residents who were aged 60 to 74 and were screened in 2012 were enrolled. The Lasso logistic regression model was used to identify the predictors for CRC. Predictive validity was assessed by the receiver operating characteristic (ROC) curve. Bootstrapping method was also performed to validate this prediction model.

CRC was best predicted by a model that included age, sex, education level, occupations, diarrhea, constipation, colon mucosa and bleeding, gallbladder disease, a stressful life event, family history of CRC, and a positive fecal immunochemical test (FIT). The area under curve (AUC) for the questionnaire with a FIT was 84% (95% CI: 82%–86%), followed by 76% (95% CI: 74%–79%) for a FIT alone, and 73% (95% CI: 71%–76%) for the questionnaire alone. With 500 bootstrap replications, the estimated optimism (<0.005) shows good discrimination in validation of prediction model.

A risk prediction model for CRC based on a series of symptoms and signs related to enteric diseases in combination with a FIT was developed from first round of screening. The results of the current study are useful for increasing the awareness of high-risk subjects and for individual-risk-guided invitations or strategies to achieve mass screening for CRC.

## 1 Introduction

There are numerous risk prediction models for colorectal cancer (CRC) in the literature,^[1,2]^ including both genetic^[3–10]^ and non-genetic models.^[11–22]^ The former only ascertains information from a small proportion of population that has inherited a genetic mutation related to several major genes, such as the MMR genes, APC, MUTYH, STK11 (LB1), BMPR1A, and PTEN. The most notable model is the MMRpro model, which considers the dominantly inherited, highly penetrant mutations (MLH1, MSH2, or MSH6).^[3]^ The non-genetic models aim to identify high-risk subjects based on non-genetic, personal, and environmental factors together with a family history of CRC. Although these models are probably used to identify subjects with a high risk of CRC susceptibility, there are still some concerns over their usefulness. Genetic models can only identify half of the familial risk of CRC,^[23]^ and the predictive accuracy of non-genetic models is modest.^[20–21]^

To increase the predictive validity of these prediction models, a history of screening or diagnosis with colonoscopy has been incorporated into the risk prediction model, according to previous studies.^[14–16]^ In addition to the well-established risk factors, adding information related to the awareness variables might help capture the risk of CRC susceptibility. This aspect can partially account for an ethnic-specific incidence of CRC worldwide because the extent of awareness may affect the detection and early treatment of CRC, as can genetic differences. Adding this information also plays an important role in individual-risk-guided community-based screening. CRC awareness is still low in Asian countries compared with Western countries. Self-reported symptoms and signs related to enteric disease may provide useful information regarding the economic influence of early CRC detection in a country with low awareness. Although these clinical symptoms and signs may not be associated with CRC, they may create an incentive for being wary of seeking medical care to detect CRC earlier.

In addition to considering the history of screening by colonoscopy, the use of a fecal immunochemical test (FIT) may provide valuable information,^[24,25]^ as a previous study suggested performing a colonoscopy to detect colorectal cancer. How to incorporate information on the history of FIT into a risk prediction model should also be considered to improve awareness.

Data from population-based screening for CRC with a questionnaire and fecal immunochemical test (QFIT), in Tianjin, offer an opportunity to test whether and how information on the personal characteristics of the symptoms and signs related to enteric diseases obtained from the questionnaire together with FIT are associated with the risk for CRC. The aims of this study were to examine the association between a constellation of demographic features and clinical correlates on the personal symptoms and signs related to enteric diseases. Those correlates that were identified as significant were used to compute the individual risk score and build a risk prediction model for colorectal cancer.

## 2 Methods

### 2.1 Study samples

We recruited 891,199 community residents aged 60 to 74 years who participated in a Tianjin community-based CRC screening program in China in 2012. Information on CRC cases consisted of 207 screen-detected CRC cases and 264 clinically detected CRC cases identified from the Tianjin Union Medical Center during 1 year of follow-up of the entire screened cohort.

### 2.2 Screening protocol and mass screening

The procedure of screening began with a questionnaire that contained personal characteristics and a constellation of clinical correlates. Those subjects who were defined as high-risk based on the questionnaire (53,631 out of 891,199) were referred to arrange further colonoscopic examinations. Those who were defined as low-risk based on the questionnaire were suggested to undergo a FIT (ABON, China). Due to the evaluation purpose of the pilot phase, a proportion of subjects returning a high-risk questionnaire also had a FIT performed to provide information for evaluating the sequential design. Since FIT is expected to be more accurate in detecting CRC than the questionnaire is, we had approximately one-third of the subjects identified as high-risk based on the questionnaire (20,633 out of 53,631) undergo a FIT and approximately two-thirds of the low-risk subjects based on the questionnaire (533,449 out of 891,199) undergo a FIT. Note that only a fraction of positive subjects was referred for colonoscopy.

### 2.3 Questionnaire

A structured questionnaire that included personal characteristics and a constellation of clinical correlates was provided during a screening activity. We examined the demographic information, including age, sex, marital status, education level, and occupation. We also measured the clinical correlates based on the following nine questions: chronic diarrhea history; chronic constipation history; mucus or blood stool history; chronic appendicitis or appendectomy; chronic gallbladder disease or gallbladder surgery history; stressful life event over the past 2 decades; cancer history; colon polyps history; or family history of colorectal cancer among first-degree relatives. The questionnaire was performed by trained public health nurses.

Subjects who had any first-degree relatives with CRC cancer, who had ever been affected by polyps or cancer or who had ≥2 of the following clinical syndromes, that is, chronic constipation, chronic diarrhea, bloody mucus, history of negative life events, history of chronic appendicitis or appendectomy, history of chronic gallbladder disease or gallbladder surgery, were defined as high-risk subjects. Those who were defined as high-risk subjects based on the questionnaire were referred for further colonoscopic examination. Those who were defined as low-risk subjects based on the questionnaire were suggested to undergo a FIT. Subjects with positive findings on the FIT were also referred for colonoscopic examination.

### 2.4 Immunochemical test

Fecal samples were obtained from 550,318 subjects at their home using the collection kit provided by the manufacturer (ABON, China). Participants were asked to collect 10 to 50 mg of stool and send it to the community hospitals. No specific dietary restriction was stipulated. All tests were processed at the laboratory within 8 hours after collection. According to the manufacturer's instructions, this qualitative test is considered positive when the sample is positive for hemoglobin. The results were reported by the central laboratory in a qualitative manner (positive and negative). Finally, 4% of the stool samples were randomly selected for a re-test quality control.

### 2.5 Ethical consideration

The original research protocol was reviewed and approved by the ethical review committee of Tianjin Union Medical Center. The program performs annual recruitment screenings, which are approved by the local ethical committee in the Health Bureau of Tianjin City. These approvals include data linkage systems and strict maintenance of participant confidentiality. Because the personal identification numbers for the datasets were encoded, the privacy and confidentiality of patients were ensured by obscuring the links between datasets.

### 2.6 Statistical analysis

A Lasso logistic regression model was used to select potential predictors of CRC and to estimate the regression coefficients for the relationships between the predictors and CRC. The Hosmer–Lemeshow goodness-of-fit test was used to determine whether the prediction model was correctly specified. The calibration plots have the predicted probabilities for groups defined by ranges of individual predicted probabilities (10 groups of equal size) on the *x*-axis, and the mean observed outcome on the *y*-axis. These plots are graphical illustrations of the Hosmer–Lemeshow goodness-of-fit test. The receiver operating characteristic (ROC) curve analysis was applied for prediction. We determined the predictive ability for the prediction model by examining the area under the curve (AUC) using a non-parametric method such as the Mann–Whitney *U* test. We examined the models with questionnaire-based or FIT predictors. We also examined the model with both the questionnaire and FIT. The bootstrap method was adopted to validate the prediction model. We first developed our prediction models with the total sample and then generate a bootstrap sample by sampling n individuals with replacement from the original sample. The sample size varied according to the number of events per variable (EPV). In order to develop an adequate predictive model, it has been suggested that EPV should be at least 10.^[26]^ Thus, we present detailed results for EPV values starting from 5 to 80. The apparent performance was determined on random samples from the data set for EPV 5, 10, 20, 40, and 80, respectively. Simulations were repeated 500 times.

## 3 Results

### 3.1 Determination of predictors from model selections

The relevant factors included demographic factors (age, sex, education, and occupation); personal disease history of diarrhea, constipation, colon mucus and bleeding; appendicitis; occurrence of gallbladder disease; history of colon cancer and polyps; stressful life events; and family history. Table 1 shows the distribution of selected variables for non-CRC and CRC among the participants. The unadjusted association between each candidate predictor and the risk of colorectal cancer are given in Table 2. Table 2 also shows the estimated regression coefficients for the associations between each factor and the risk for colorectal cancer, after adjusting for confounding factors.

**Table 1:**
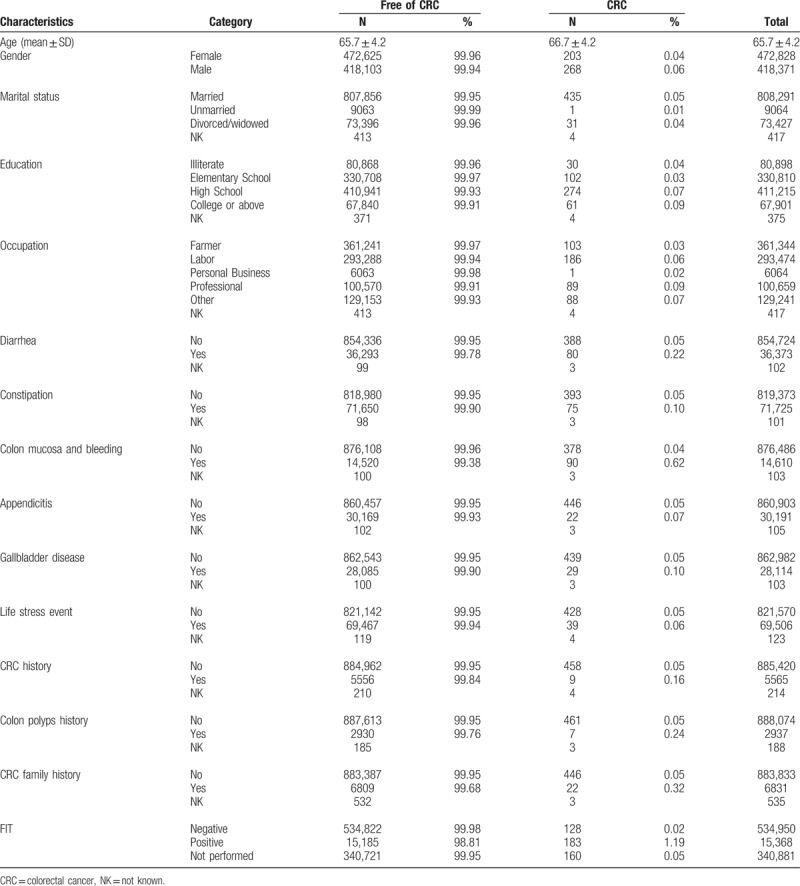
Distribution of characteristics for free-of-CRC versus CRC participants.

**Table 2:**
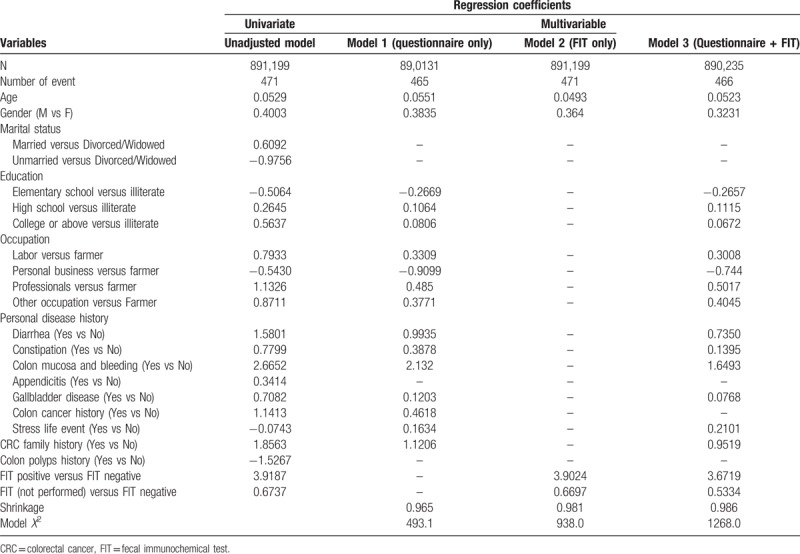
Regression coefficients for predictors by model selection.

The selected predictors, including age, sex, education level, occupation, diarrhea, constipation, colon mucus and bleeding, gallbladder disease, personal colon cancer history, stressful life event, and a CRC family history, were included in model 1 (questionnaire only model). It is very interesting to note that those subjects who did not undergo a FIT were at a greater risk than were those who had undergone a FIT when the high-risk subjects, as defined by the questionnaire, were considered a separate risk group. Those subjects with a positive FIT result had an extremely high risk for CRC compared with those who had negative FIT results (model 2).

In addition to FIT, the predictors determined by model selection, including age, sex, education level, occupations, diarrhea, constipation, presence of colonic mucus and bleeding, gallbladder disease, stressful life event, and CRC family history, were included in model 3 (questionnaire plus FIT model).

Table 3 shows the estimated results for the variables relevant to the use of FIT, after adjusting for age and sex. The differences were all statistically significant (*P* < .0001).

**Table 3:**
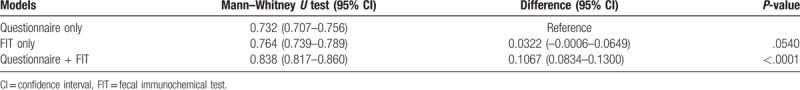
Comparison of areas under the curve (AUCs) for conventional risk predictors, FIT, or a combination.

### 3.2 Risk score for CRC

According to regression coefficients estimated from the logistic regression model (Questionnaire + FIT) as shown in Table 2. The individual risk score was built while considering FIT using the following equation:

Risk Score = 0.5 × Age + 3 × Gender (M:1;F:0) – 2.7 × Education (Elementary school:1;Illiteracy:0) + 1.1 × Education (High school:1;Illiteracy:0) + 0.7 × Education (College or above:1; Illiteracy:0) + 3.0 × Labor (1 or 0) – 7.4 × Personal Business (1 or 0) + 5 × Professionals (1 or 0) + 4 × Other Occupation (1 or 0) + 7.4 × Diarrhea (1 or 0) + 14.0 × Constipation (1 or 0) + 16.5 × Colon Mucosa (1 or 0) + 7.7 × Gallbladder disease (1 or 0) + 2.1 × Stress life event (1 or 0) + 9.5 × CRC Family History (1 or 0) + 36.8 × FIT Positive (1 or 0) + 5.4 × FIT Not Perform (1 or 0).

**Figure 1:**
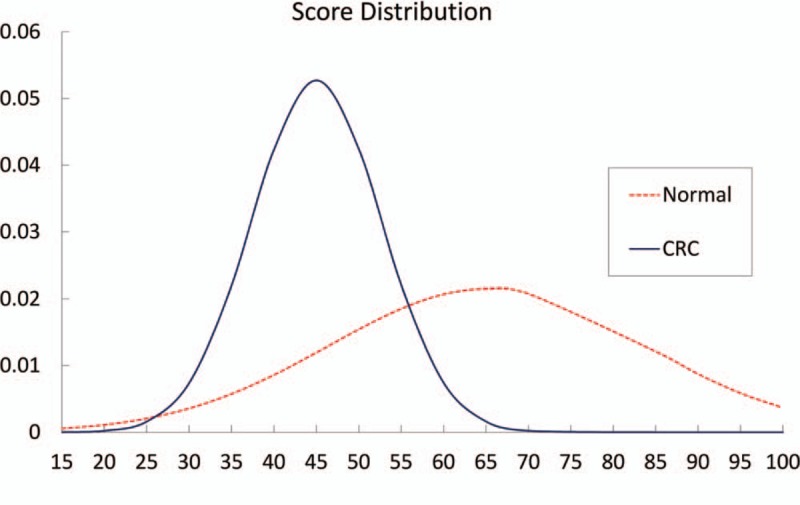
Risk score distribution for Tianjin population.

The distribution of the risk scores between free-of-CRC and CRC subjects is shown in Fig. 1. The difference between the groups was statistically significant (*t* = –23.38, *P* < .0001).

### 3.3 Calibration

**Figure 2:**
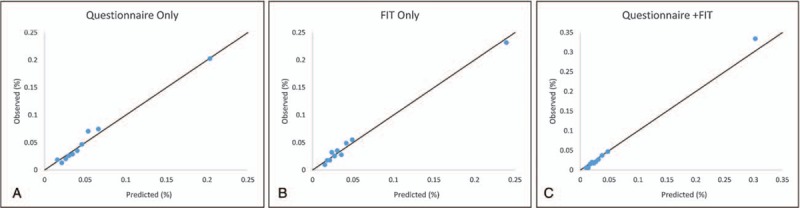
Calibration plots.

The comparison of the observed and predicted probabilities for the models according to the Hosmer–Lemeshow test is provided. The calibration plots are presented to reflect the agreement between observed outcomes and predictions (Fig. 2). The figures show perfect moderate calibration. The calibration lies on or around a 45° line of the plot. All of three developed prediction models show the good model calibration (Questionnaire only model: χ^2^ = 11.77; *P* = .1617, FIT only model: *χ*^2^ = 9.38; *P* = .3116, Questionnaire plus FIT model: *χ*^2^ = 11.92; *P* = .1549).

### 3.4 ROC analysis

**Figure 3:**
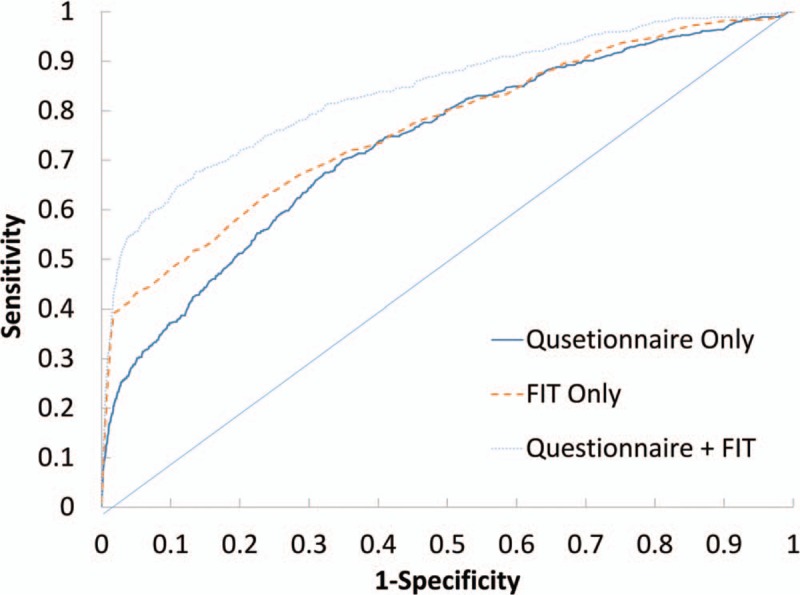
Receiver operating characteristic curves for different modalities of CRC screening. CRC = colorectal cancer.

The prediction models with significant correlates from the questionnaire plus FIT, FIT only, and the questionnaire only are also presented with ROC curves. Figure 3 shows that the questionnaire correlates in combination with FIT performed best among the 3 modes. The AUC (*c*-index) for the questionnaire with FIT was 0.838 (95% CI: 0.817–0.860), followed by 0.764 (95% CI: 0.739–0.789) for FIT only, and 0.732 (95% CI: 0.707–0.756) for the questionnaire only. A comparison between the full model and the model with FIT only, based on the non-parametric Mann–Whitney *U* test, is shown in Table 3.

### 3.5 Validation of prediction model

The optimism is the difference between model performance in the bootstrap sample and in the original sample. In model 3 (Questionnaire + FIT), with 500 bootstrap replications, the estimated optimism was 0.00411 for EPV = 5, and the apparent performance for *c*-index of 0.8429 (=0.8388 – 0.00411) shows good discrimination. With large sample size (EPV = 40 or 80), a reduction in optimism was found. The mean apparent *c*-index were 0.8438, 0.8407, 0.8372, and 0.8387 for EPV = 10, EPV = 20, EPV = 40, and EPV = 80, respectively. The 0.8387 of *c*-index for EPV = 80 was most close to the *c*-index of 0.8388 in original sample. Similar findings were observed in model 1 (Questionnaire only) and model 2 (FIT only). The mean apparent *c*-index were 0.7395 and 0.7315 for EPV = 5 and EPV = 80 in model 1. The mean apparent *c*-index were 0.7699 and 0.7639 for EPV = 5 and EPV = 80 in model 2.

## 4 Discussion

In contrast to previous risk prediction models that either incorporate dominant and high-penetrance genes into a genetic model or include personal characteristics and environmental risk factors in the non-genetic model, our risk prediction model for CRC is specific and considers the history of clinical symptoms and signs of bowel disease together with the FIT results. A risk score was developed after training regression coefficients as clinical weights to assess the influence that each correlate has on the risk for CRC by using empirical data from the QFIT program in Tianjin, China.

We found that the combination of information obtained from the questionnaire with FIT resulted in a good prediction for the risk of CRC, with an AUC up to 84% when using the non-parametric Mann–Whitney *U* method. The FIT information alone gives a lower prediction that is equivalent to that obtained when using the questionnaire alone, with respect to AUC (76% vs 73%); however, this difference lacks statistical significance based on the Mann–Whitney *U* test.

The factors associated with CRC awareness could be considered predictors for predicting the risk of CRC. Such a prediction model might be useful for alerting someone with clinical symptoms or signs and thereby detecting CRC earlier.

The same logic is applied to administering a FIT to improve patients’ awareness and detect early CRC cases. These clinical correlates may be a reflection of the proxy variables for residual familial risk, after making allowances for a family history of CRC.

Our prediction models were validated using bootstrapping method. The apparent performance for logistic regression model in data sets with 5 to 80 events per variable (EPV) with 500 bootstrap repetitions was estimated for internal validation. The low estimated optimism indicates a good discrimination for a given EPV value in our analysis. The apparent performance was more close to the performance in the total dataset while increasing the sample size (EPV = 80).

In light of these risk prediction models, providing an individual-guided risk approach is an alternative and efficient way to achieve the goal of mass screening for early detection of CRC in Asian countries that have a low or intermediate incidence rate. For example, the risk score can be built from data obtained in the first screening to develop a risk prediction model. This prediction model can then be applied in subsequent screenings by offering an individual-risk-guided invitation to a large population-based screening program.

The greatest strength of our risk prediction model is that it was developed by using large community- and population-based screening data. The threat to validity due to the selection bias that is inherent when using consecutive cases series from hospitals, as is usually found in case-control studies or clinical studies,^[12]^ can be ameliorated. Large community- and population-based studies have also gained sufficient statistical power for building a risk prediction model for CRC, as can be seen by the narrow confidence interval for the AUC of the ROC curves.

The main limitation is that we have not considered incorporating information into the genetic model about carrying a genetic mutation related to several major genes (such as the MMR genes, APC. MUTYH, STK11 [LB1], BMPR1A, and PTEN).^[3]^ The binary property of family history is not adequate for capturing familial risk without considering the age of onset of CRC among relatives. Our risk prediction model is therefore not adequate for predicting CRC in association with familial risk. This should be considered when using family pedigree data obtained from, for example, a Keelung community-based integrated screening study.^[27]^

In conclusion, we have developed a CRC risk prediction model based on a series of symptoms and signs related to enteric diseases in combination with FIT. Such a risk prediction model is useful for improving the awareness of high-risk subjects and for individual-risk-guided invitation or strategies to achieve mass CRC screening.

## Author contributions

**Conceptualization:** Li-Zhong Zhao, Dong-Wang Ma, Sam LI-SHENG Chen, Xi-Mo Wang.

**Data curation:** Dong-Wang Ma, De-Zheng Wang, Lei Shi, Hong-Lei Wang, Mo Dong, Shu-Yi Zhang, Lei Cao, Wei-Hua Zhang, Xi-Peng Zhang, Qing-Huai Zhang, Lin Yu, Hai Qin, Xi-Mo Wang.

**Formal analysis:** Li-Zhong Zhao, Sam LI-SHENG Chen.

**Investigation:** Li-Zhong Zhao, Dong-Wang Ma, De-Zheng Wang, Lei Shi, Hong-Lei Wang, Mo Dong, Shu-Yi Zhang, Lei Cao, Wei-Hua Zhang, Xi-Peng Zhang, Qing-Huai Zhang, Lin Yu, Hai Qin, Xi-Mo Wang.

**Methodology:** Li-Zhong Zhao, Sam LI-SHENG Chen.

**Project administration:** Dong-Wang Ma, Xi-Mo Wang.

**Supervision:** Xi-Mo Wang.

**Validation:** Sam LI-SHENG Chen.

**Writing – original draft:** Wen Li.

**Writing – review & editing:** Li-Zhong Zhao, Xi-Mo Wang.
